# Closing of Wards at the London Hospital

**Published:** 1921-06-04

**Authors:** 


### The Hospital

The Workers' Newspaper of Administrative Medicine and Institutional Life, Administration, National Insurance and Health.

No. 1826, Vol. LXX. SATURDAY, JUNE 4, 1921. PRICE TWOPENCE

### CLOSING OF WARDS AT THE LONDON HOSPITAL.

Unfortunately it still remains true, at the time of writing, that the London Hospital and King's College Hospital have closed their several wards in vain, so far as invoking any fresh assistance. Yet there coincided with this regrettable decision a surprise visit from Queen Alexandra to the London Hospital, which showed that Her Majesty at least was fully alive to the gravity of the case; nor do we believe that she visited the hospital primarily because she is its President. It is only natural that the Royal Family, whose members have done so much to help the expansion of the voluntary hospitals during the past fifty years, should be more alive than most people to the present enormous difficulties. Another point to note is that Lord Knutsford's letters to the newspapers, still couched in that language of appeal which he has so often used successfully, have not, so far, excited any response equal to the need. The history of institutional appeals would probably show more remarkable ups and downs than that of most activities. It is not long since the Red Cross had only to raise its finger to receive whatever it cared to ask, yet its attempt last year on exactly similar lines and over the most exalted signature was not conspicuous for its success.

We have no official particulars before us of the exact financial position of the London Hospital, but it is understood that the hospital costs £4,000 a week to run, and the present income is £2,500. It seems, however, that, in view of the fact that Lord Cave's Committee's report is promised by the end of this month, when, doubtless, some immediate means of relief will be suggested, the drastic step of closing seven wards in what is practically the only general hospital in the East End of London might have been delayed for a few weeks. Closing a large part of a hospital and getting rid of a trained and valued staff is a very serious step, and one which cannot be retrieved without a large amount of trouble and expense; with this consideration in mind it is difficult to appreciate the wisdom of the step to be taken at the Italian Hospital, where the work is to be closed down for one month. Are salaries and all main expenditure to continue? If so, the saving appears hardly to justify the upset caused by such drastic action.

Possibly Lord Knutsford is doubtful whether the measures of relief suggested have sufficient promise in them to justify further borrowing, although, no doubt, it would be the easiest matter in the world to secure help of this kind. Fortunately, elsewhere greater optimism is shown; the announcement by Lord Athlone that the Middlesex Hospital is obtaining at least £10,000 a year through patients' payments is an evidence that those who are responsible for hospital administration are finding means of raising revenue which have not been tried before. In connection with the Middlesex, it is stated that a possible asset is a claim on the Government, representing the difference between grants made for the treatment of naval and military patients and the actual cost of their maintenance. If this liability is admitted and met it will materially relieve the position. (*See also p.* vi.)

